# *Triticum aestivum* WRAB18 functions in plastids and confers abiotic stress tolerance when overexpressed in *Escherichia coli* and *Nicotiania benthamiana*

**DOI:** 10.1371/journal.pone.0171340

**Published:** 2017-02-16

**Authors:** Xiaoyu Wang, Linsheng Zhang, Yane Zhang, Zhenqing Bai, Hao Liu, Dapeng Zhang

**Affiliations:** College of Life Sciences/State Key Laboratory of Crop Stress Biology for Arid Areas, Northwest A & F University, Yangling, China; Huazhong University of Science and Technology, CHINA

## Abstract

WRAB18, an ABA-inducible protein belongs to the third family of late embryogenesis abundant (LEA) proteins which can be induced by different biotic or abiotic stresses. In the present study, *WRAB18* was cloned from the *Zhengyin 1* cultivar of *Triticum aestivum* and overexpressed in *Escherichia coli* to explore its effects on the growth of *E*. *coli* under different abiotic stresses. Results suggested the enhanced exhibition of tolerance of *E*. *coli* to these stresses. Meanwhile, the *WRAB18*-transgenic tobacco plants were obtained to analyze the stress-related enzymatic activities of ascorbate peroxidase (APX), peroxidase (POD) and superoxide dismutase (SOD), and to quantify the content of malonaldehyde (MDA) under osmotic stress, high salinity, and low and high temperature stress. The activities of APX, POD and SOD in the transgenic tobacco lines were higher while the content of MDA was lower than those of WT lines. Moreover, plastid localization of WRAB18 in *Nicotiana benthamiana* plasma cells were found fusing with GFP. In addition, purified WRAB18 protein protected LDH (Lactate dehydrogenase) enzyme activity *in vitro* from various stress conditions. In brief, WRAB18 protein shows protective action behaving as a “molecular shield” in both prokaryotic and eukaryotic cells under various abiotic stresses, not only during ABA stress.

## Introduction

Abiotic stresses were key restriction factors during the growth and production of crops. Plants have developed numerous defense mechanisms to protect themselves under complex growth conditions through long-term evolutionary processes. These mechanisms are found at the cellular and molecular levels, such as changes in membrane lipid composition, production of new protein polymers, increased contents of sugars, organic acids, soluble proteins, proline, and ABA, and other corresponding changes necessary to resist or avoid adverse conditions [1]. LEA proteins represent one of these adaptions and are believed to play an important role in adverse physiological regulatory processes, although these proteins are common in plants under abiotic stress conditions [2]. However, their specific physiological and biochemical functions remain unclear. Therefore, further studies on the mechanisms of LEA proteins are required [3].

Late embryogenesis abundant (LEA) proteins were first identified in late-stage mature cotton seeds by Dure and Croudh in 1981 [4]. Since then, they have been identified in many other species including higher plants, such as wheat [5], rice [6], maize [7], barley [8], and bean [9], and in primary species, such as *Artemia franciscana* [10], nematodes [11], and bacteria [12]. LEA proteins are widely distributed in seeds, roots, stems, and leaves of plants [13]. They are classified into seven groups according to the amino acid sequences and conserved motifs [14]. Group 3 LEA proteins have been characterized based on an 11-amino acid motif, TAQAAKEKAGE [15, 16]. They exist in a randomly coiled structure in plant cells and have a high percentage of hydrophilic amino acids, such as glycine, serine, and threonine, which form a reversible α-helix under dehydrated conditions and subsequently revert back to the randomly coiled structure upon rehydration [17, 18]. The hydrophilic α-helix may be related to protein-protein and protein-lipid interactions during stress situations [19], that potentially maintains membrane stability and integration under stress [20, 21]. However, the functions discussed above are inferred based on existing observations, and no direct evidence is available *in vivo* to support these conclusions. Therefore, further studies are required to explore the specific functions.

At this point, numerous of novel group 3 LEA proteins have been isolated and identified in different species. Twenty-three LEA genes were identified from the *Pinus*. *tabuliformis* by Gao et al. (2016), six of them belong to the third group of LEA family, which can improve the resistance of *E*. *coli* strains against heat and salt stresses when they are expressed abundantly in *E*. *coli* [22]. Four novel genes identified from the sorghum genome were classified into LEA3A and LEA3B subgroups according to the conservative specific motifs of group 3 LEA proteins [23]. Since LEA proteins have been discovered, and sequence features have been analyzed, their specific functions were explored. Several LEA proteins were isolated, analyzed, and detected. LEA proteins have been transferred into different cell types to examine the contributions they make to biological cells under stress. ZmLEA3, located in both the cytosol and nucleus, binds to metals such as Fe^3+^, Mn^2+^, Zn^2+^, and Mn^2+^ under osmotic and oxidative stress. Further experimental investigations showed that overexpression of ZmLEA3 in yeast (GS115) and tobacco (*Nicotiana benthamiana*) improved the tolerance to biotic stress [7]. The protective effect of OsLEA4 on the growth of *E*. *coli* during stress was confirmed, and it was showed that OsLEA4 functions as a protective factor in prokaryotic cells [24]. Furthermore, *HVA1*, a group 3 LEA gene, was cloned from barley and expressed in rice, data showed that transgenic rice had a strong tolerance to salt stress and dehydration [25, 26]. Moreover, after inserting *HVA1* into the wheat genome, the transgenic wheat showed the increase in biomass and water usage efficiency under hydrated conditions [27]. Cells expressing Mg3-GFP were found to show reduced cell shrinkage effects during dehydration [28]. Overall, numerous studies have shown that LEA proteins play an important role in protecting plants from damage caused by drought, low or high temperature, or salinity stress.

In previous reports, the full-length sequence of *WRAB18* (accession no. AB115914) was obtained, and its corresponding amino acid sequence was analyzed using the BLAST algorithm. It belongs to the 3A subgroup of group 3 LEA protein family harboring the conserved domain [14, 29]. WRAB18 is a hydrophilic protein with high amino acid quite identical to WRAB19 in wheat [30] and HVA1 in barley [31]. It has been reported that WRAB18 is induced by ABA treatment and responds to ABA stress, but its other roles in abiotic stress remain unclear [32].

To further explore the function of WRAB18, we cloned its cDNA sequence from the wheat cultivar *Zhengyin 1*. In both prokaryotic and eukaryotic species, *in vivo* and *in vitro* experiments were performed to verify the response of WRAB18 to other stress factors (excluding ABA-related inductivity), and the subcellular localization and protective effects towards other enzyme activity under various stresses were examined.

## Materials and methods

### Plant materials and stress treatments

Winter wheat (*Triticum aestivum* cultivar *Zhengyin 1*) was used throughout this study. Seeds were preserved by the State Key Laboratory of Crop Stress Biology for Arid Areas, Northwest A & F University, Yangling, China. Seeds were first soaked in distilled water overnight and treated with 70% alcohol for 5 min, washed six times with sterile water, and then placed on culture dishes over two layers of wetted filter paper under 200 μE m^-2^s^-1^ light with the light/dark cycle conditions of 16/8 h at 28°C for 2 weeks. Shoots were watered, and the double-layered filter paper was drenched to avoid anaerobic stress caused by a flooding of water.

The *Nicotiana benthamiana* seeds used in the subcellular localization experiment were obtained from College of Life Sciences, Northwest A & F University, Yangling, China and sown in soil mixed with vermiculite at a 1:1 ratio for 4 weeks in a greenhouse under 16-h photoperiod at 25°C and were watered with deionized distilled water (ddH_2_O).

### Amplification of the *WRAB18* gene and expression of the WRAB18 protein

Total RNA was extracted from wheat (*Triticum aestivum* cultivar *Zhengyin 1*) using Trizol reagent (Invitrogen). First-strand cDNA synthesis was performed using 500 ng total RNA, and oligo dT and random primers were used according to the manufacturer’s instructions of PrimeScript RT reagent Kit with gDNA Eraser (TaKaRa). The open reading frame of *WRAB18* was amplified from cDNA using gene-specific primers (S1 Table) based on the sequence from NCBI (accession no. AB115914). The products were cloned into pMD18-T vectors (TaKaRa) and sequenced. Physicochemical property analysis of WRAB18 was done based on the following web site http://web.expasy.org/protparam/.

The open reading frame of *WRAB18* was cloned from cDNA using gene-specific primers with the appropriate restriction enzyme digestion sites for *Eco*RⅠ in the forward primer and *Hind* Ⅲ in the reverse primer (S1 Table). The products were ligated into pET28a (Novagen), and sequencing analysis verified transformation of the *WRAB18*-pET28a recombinant plasmid into *Escherichia coli* strain BL21 (DE3) competent cells (TransGen Biotech). WRAB18 was expressed with an N-terminus 6×His tag, a thrombin cleavage site and a T7 tag, since the stop codon was designed in the reverse primer to avoid appending a C-terminal 6×His tag.

The positive strains were grown at 37°C in Luria-Bertani (LB) agar medium supplemented with kanamycin (50 μg/mL). After 3–4 h of growth until the OD_600_ reached 0.4–0.5, isopropylthio-β-D-galactoside (IPTG) was added to the cultures to a final concentration of 1.0 mM and grown at 37°C for 12 h to induce expression of the target protein. After boiling the bacterial suspension at 95°C for 3 mins, the supernatant was examined by 12% SDS-PAGE, and protein bands were stained with Coomassie Brilliant Blue R-250 [33].

Immunoblotting analysis was performed to confirm the expression of WRAB18. Proteins were transferred to polyvinylidene fluoride (PVDF) microporous membrane at 80mA for 50 min. After washing three times for 10 min each time with 20 ml of 1×TBST buffer (20 mM Tris-Hcl, 200 mM NaCl, 0.1% Tween 20, pH7.5), the membrane was blocked in a 5% nonfat dried milk solution for 1 h. Incubation with anti-His-tag rabbit polyclonal antibody (CWBIOTECH) raised against recombinant protein was performed overnight at 4°C. Washed three times for 10 min each time with 20 ml of 1×TBST buffer, the membrane were incubated for 1 h at room temperature with the secondary antibody (goat anti-rabbit IgG; Beyotime) and washed as above, one time more for 10 min with 20 mL of 1×TBS buffer (20 mM Tris-Hcl, 200 mM NaCl, pH7.5). Then membrane was stained with freshly prepared developing buffer (200 μl NBT solution and 200μl BCIP solution in 5 mL alkaline phosphatase buffer) until signals were clearly visible. The reaction was stopped by washing the membrane in deionized distilled water (ddH_2_O).

### Assays for abiotic stress tolerance of *E*. *coli* transformants

Stress tolerance assays were performed to detect the effect of WRAB18 on *E*. *coli* viability under adverse conditions. The transformant harboring empty pET28a plasmid was used as control. BL21/WRAB18 and BL21/pET28a cultures, as well as IPTG induction, were prepared as described above. The concentration of induced cultures in liquid LB was adjusted to an OD_600_ of 1.0, after which 1 mL was inoculated into 50 mL fresh liquid LB with kanamycin (50 μg/mL) and IPTG (1.0 mM) in 250 mL flasks, and the initial concentration was adjusted to the same OD_600_ for each stress. In order to measure the salinity tolerance of transformed *E*. *coli* cells, the liquid LB was supplemented with an additional 500 mM NaCl. On the other hand, 800 mM mannitol was added to the liquid LB to detect the drought tolerance of *E*. *coli* cells. NaCl and mannitol was dissolved in the liquid LB medium without changing the bacterial culture volume. Both of the two bacterial suspensions were developed at 37°C and 200 rpm. For the thermophylactic experiments, samples were transferred to 45°C, while cells were cultured at 28°C and 200 rpm for the cryophylactic assays. Recombinant BL21/WRAB18 and BL21/pET28a in the induced cultures in liquid LB supplemented with kanamycin (50 μg/mL) and IPTG (1.0 mM) were cultured without any stressor at 37°C and 200 rpm. At each time, 3 mL of culture were used to measure the OD_600_ using a spectrophotometer. Each experiment was performed in triplicate.

### Transgenic tobacco overexpression assay

The *WRAB18* gene was amplified using primers containing *Xba*Ⅰ and *Sac*Ⅰrestriction enzyme sites (S1 Table). The cDNA was then digested with XbaⅠ and SacⅠ, purified, and ligated to linearized pBI121 binary vector (Clontech) containing the powerful *Cauliflower mosaic virus* (CaMV) 35S promoter and the kanamycin resistance gene. 10 ng of the recombinant plasmid was added into 50 μL *Agrobacterium tumefaciens* (GV3101) competent cells in 0.5 mL eppendorf tube and mixed by tapping the tube. The mixture was transferred to a pre-chilled (on ice) electroporation cuvette with 2 mm gap sizes. Electroporation cuvette was put in the holder of MicroPulser Electroporator (Bio-Rad) adjusting the gene pulser unit “Set Volts” setting to 2.5 kV and the “CAP” setting to 25 μFD. The resistance was set to 400Ω on the pulse controller unit, and the time constant was ~9 msec. After being incubated on roller drum at 28°C for ~2 hours, 200 μL of culture was plated on selective medium. Positive strains were used to infect tobacco leaves, which were cut into small pieces (2 × 2 cm). We obtained callus by tissue culture on solid MS medium. The tranformantion of *Nicotiania benthamiana* was performed using the leaf disc method [34]. Buds were placed in MS medium containing 100 mg/L kanamycin to select positive plants. After growing for 2 months, they were transferred to soil until the seeds were harvested.

Semi-quantitative RT-PCR was performed using 4-week-old transgenic tobacco leaves to identify overexpressing transgenic tobacco plants. The expression of *β-actin* gene was used as a control. A total of 10 leaves from 10 lines were analyzed for the expression of the *WRAB18* gene from genomic DNA and compared with levels in WT plants.

### Experiments assessing the growth and physiological parameters of transgenic tobacco

We observed phenotypic differences between transgenic and wild-type (WT) plants. We soaked transgenic and WT tobacco seeds in distilled water for 2 days and grew them on specific Murashige and Skoog (MS) solid medium containing 100 mM and 200 mM mannitol and 100 mM and 200 mM NaCl for drought and high salinity stress conditions, respectively. As a control, seeds were cultivated on plates on MS medium in a tissue culture room. For temperature stress, plates were cultured under low (18°C) and high (40°C) temperatures. After 14 days of growth under different conditions, phenotypic differences were observed and analyzed statistically. For the experiment of germination rate, root length, and survival rate, the number of samples was 100.

Meanwhile, the transgenic and WT seeds were sowed in soil for 4 weeks; the seedlings were treated with different stresses (osmotic stress, high salinity, and low and high temperatures) for 48 h. Leaves were collected to perform enzyme activity measurements of ascorbate peroxidase (APX), peroxidase (POD) and superoxide dismutase (SOD), and the contentration of malonaldehyde (MDA) was also quantified. [35].

### Lactate Dehydrogenase (LDH) enzyme activity assay

WRAB18 appended with a 6×His tag was expressed by the *E*. *coli* system and purified using His [Ni^2+^] resin. The LDH enzyme (EC1.1.1.27, rabbit muscle lactate dehydrogenase) from Sigma (USA) was diluted to a final concentration of 10 μg/mL in sodium phosphate solution, pH 7.4. Bovine serum albumin (BSA, Sigma, USA) and sucrose solution, as a control, were prepared at 20 μg/mL of 20 μL with sodium phosphate (pH 7.4), which was the same to WRAB18. The 20 μL LDH solution was mixed with the same volumes of BSA, sucrose, and WRAB18 protein solution, respectively, then provided to various stress treatments. These reaction systems were then exposed to high (45°C) and low (0°C) temperature for 30 min and 12 h, respectively. Drought treatment was performed using a dryer (Vaccubrand, Germany), and samples were dehydrated to 70% and rehydrated to the original volume. For high salinity treatment, samples were treated with reaction buffer containing 200 mM NaCl for 2 h. LDH in solutions with no treatment was used as control. Before measuring the LDH activity, the samples were diluted to reach an enzyme concentration of 0.5μg/mL, then a total of 750 μL of reaction mix containing 10 mM pyruvic acid, 0.2 mM NADH, and 10 mM sodium phosphate, pH 7.4, was added to each reaction system to ensure that the concentration of NADH in each system was 0.13 mM. Reaction mixture was added to the 12 well plates, and the absorbance at 340 nm was measured with SpectraMax M2 Molecular Devices over 3 min at different time intervals according to the different treatments. Each reaction was repeated three times, and triplicate data were analyzed using ancillary software (SoftMax Pro, Molecular Devices, USA).

### Real-time quantitative PCR analyses

Real-time quantitative PCR was performed using the SYBR PrimeScript^™^ RT-PCR Kit (TaKaRa) at 25μL volume on the CFX96TM Real-time system (Bio-Rad, USA).

The cDNA templates for this experiment were prepared from different tissues (roots, stems and leaves) of two-leaf-period wheat seedlings. Primers were shown in S1 Table, the expression of *β-actin* gene was used as control. PCR thermal cycles were set at 95°C, 30 s for 1 cycle; 95°C, 5 s; 60°C, 30 s and 72°C, 30 s for 40 cycles; 72°C, 10 min for 1 cycle. Each reaction was replicated three times and relative-fold expression was analyzed according to the Livak method [36].

### Transient expression in protoplasts of *Nicotiana benthamiana*

A reformed vector, named pA7GFP, harboring the CaMV 35S promoter and *GFP* gene sequence was used in this study [37, 38]. The full length of *WRAB18* ORF sequence, containing the *Xba*Ⅰ restriction enzyme site at the 5′ end and the *Sma*Ⅰ restriction enzyme site at the 3′ end, was amplified (S1 Table). The PCR product was digested using these two restriction endonucleases, and the target fragments harboring the GFP gene sequence at the 5′ end were inserted into pA7GFP vectors. The identified positive recombinant vectors and plastid-localization marker plasmid (pt-rk CD3-999) [39] were transferred into the *Agrobacterium tumefaciens* GV3101 strain by electric shock. These two positive bacterial strains were cultivated in 100 mL LB liquid medium under antibiotic selection (50 mg/L rifampicin + 50 mg/L kanamycin) at 28°C for 24 h and then centrifuged at 2616 g-force for 5 min to collect cells. After resuspension in 5 ml Agromix (10 mM MgCl_2_, 10 mM MES/KOH pH 5.6, and 150 μM acetosyringone), the bacteria were cultured in dark at 28°C for 4 h. The samples were diluted to an OD_600_ of 1.0, after which the two samples were mixed. The mixture was extracted using a needleless syringe and infiltrated into three fully stretched blades of plants of 4 weeks old. The strains with empty pA7GFP plasmids were used as a control. After 2-day cultivation, the infiltrated leaves were digested using enzyme solution containing cellulase and mecerozyme R10 to obtain protoplasts according to the referred protocol [40]. The fluorescence analysis was performed using an inverted confocal microscope (Nikon A1R). The GFP fusions were excited with a 488 nm argon laser and detected using a 505–530 nm band-pass emission filter, mCherry fusions were excited using a 561 nm laser and detected using a custom-made 595–620 nm band-pass emission filter.

### Statistical analysis

Data analysis were done using GraphPad Prism version 5.0 (USA) with a t-test or ANOVA. Significant differences were indicated as a *P*-value less than 0.05.

## Results

### Cloning and sequence analysis of *Wrab18*

To determine the function of WRAB18 under various abiotic stresses, the nucleic acid sequences of *WRAB18* (GeneBank accession no. AB115914) were cloned from *Triticum aestivum*. cultivar *Zhengyin 1*. The *WRAB18* cDNA contained an open reading frame of 510 bp encoding a protein of 169 amino acids with a predicted molecular weight of 17.53 kDa and a pI of 5.95. The WRAB18 protein (GeneBank accession no. BAC80266.1) is classified as a group 3 LEA protein, similar to other known group 3 LEA proteins. The amino acid sequence of WRAB18 showed similar structural characteristics, along with the conserved domain. Multiple sequence alignment was performed using the MEGA5.0 software and the final figure was exported by ESpript 3.0 (http://espript.ibcp.fr/ESPript/cgi-bin/ESPript.cgi) (S1A Fig). In addition, WRAB18 was found to be rich in alanine (20.7%), lysine (11.8%), and threonine (12.4%) but lacking cysteine, proline, and tryptophan. The 3D structure of the wheat WRAB18 protein was predicted to show the structural domains of alpha-helixes in figure S1B with PHYRE2 at http://www.sbg.bio.ic.ac.uk/phyre2/html/page.cgi?id=index. (S1B Fig). WRAB18 protein was expressed in the *E*.*coli* and examined by 12% SDS-PAGE stained with Coomassie Brilliant Blue R-250 (S2A Fig). Immunoblotting analysis was performed to confirm the expression of WRAB18 (S2B Fig).

### Overexpression of WRAB18 enhanced the growth of *E*. *coli* under stress

Many studies used the *E*. *coli* protein expression system to obtain information on the activities of target proteins in cells, because this system is convenient, efficient, and simple to operate [41]. To examine the function of WRAB18 in prokaryotic cells *in vivo* under different stresses, *E*. *coli* cells either overexpressing WRAB18 or harbouring an empty vector control was cultured under five different conditions (37°C, with 500 mM mannitol and 500 mM NaCl at 28°C and 45°C), respectively. The bacterial solution of BL21/pET28a and BL21/WRAB18 was cultured at 37°C as a control.

**Fig 1:**
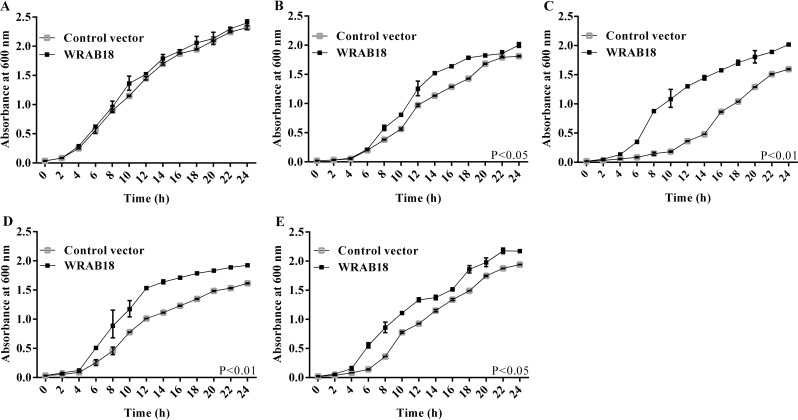
Growth curves of *Escherichia coli* cultures transformed with WRAB18 or control pET28a under four abiotic stresses. *E*. *coli* strains grown under standard culture conditions **(A)**, in medium supplemented with 800 mM mannitol **(B)** or 500 mM NaCl **(C)**, and under exposure to 28°**C (D)** or 45°**C (E)**. The OD_**600**_ was measured as an indicator of the increase in density of the liquid cultures. Each stress assay was performed three times, and statistically significant differences were analyzed using the Student’s t-test.

The OD_600_ was measured at every 2 h to generate the growth curve for *E*. *coli*, which could display the bacterial viability visually. The growth curves of bacteria showed differences under these four kinds of stress treatments. All stresses led to the inhibition to the growth of *E*. *coli*, however the extent of inhibition was quite different. As shown in Fig 1, the OD_600_ of BL21/WRAB18 was higher than that of BL21/pET28a under these four stress conditions, while the growth was consistent at 37°C (the optimal growth temperature for *E*. *coli*) (Fig 1A). BL21/WRAB18 showed better growth kinetics than those of cells carrying the empty vector under drought (Fig 1B) and at 45°C (Fig 1E). However, superior growth was observed under high salinity (Fig 1C) and cold treatment (Fig 1D), and the status under high salinity was more excellent. These growth curves of *E*. *coli* indicated that overexpression of WRAB18 increased the tolerance of *E*. *coli* strains to drought, salinity, heat, and cold, especially to high salinity and low temperature.

### Overexpression of WRAB18 increased the stress tolerance of transgenic plants

**Fig 2:**
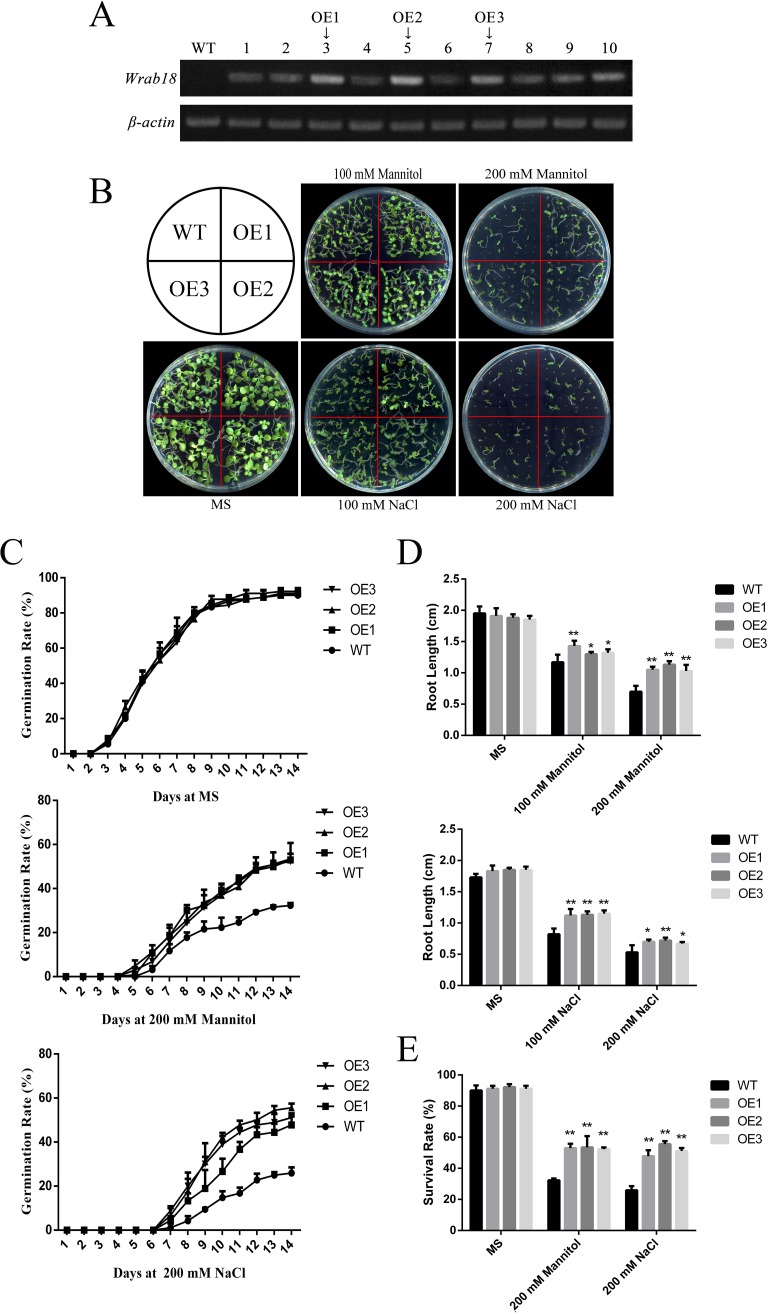
Drought and salinity tolerance in wild-type versus *Wrab18*-overexpressing *N*. *benthamiana* plants. **(A)** Identification of transgenic tobacco plants by semi-quantitative PCR. **(B)** Phenotypic discrepancy of wild-type and OE lines on MS medium containing different concentrations of mannitol and NaCl. **(C)** Germination rate of wild-type and OE lines on MS under normal conditions, 200 mM mannitol, or 200 mM NaCl. **(D)** Root length and **(E)** survival rate of wild-type and OE lines on MS under normal conditions, 200 mM mannitol, or 200 mM NaCl. Statistically significant differences were analyzed using one-way ANOVA (*p < 0.05 or **p < 0.01).

Based on semi-quantitative RT-PCR, we chose three transgenic lines, OE1 (Lane 3), OE2 (Lane 5), and OE3 (Lane 7), with the highest *WRAB18* gene expression for subsequent comparisons (Fig 2A).

**Fig 3:**
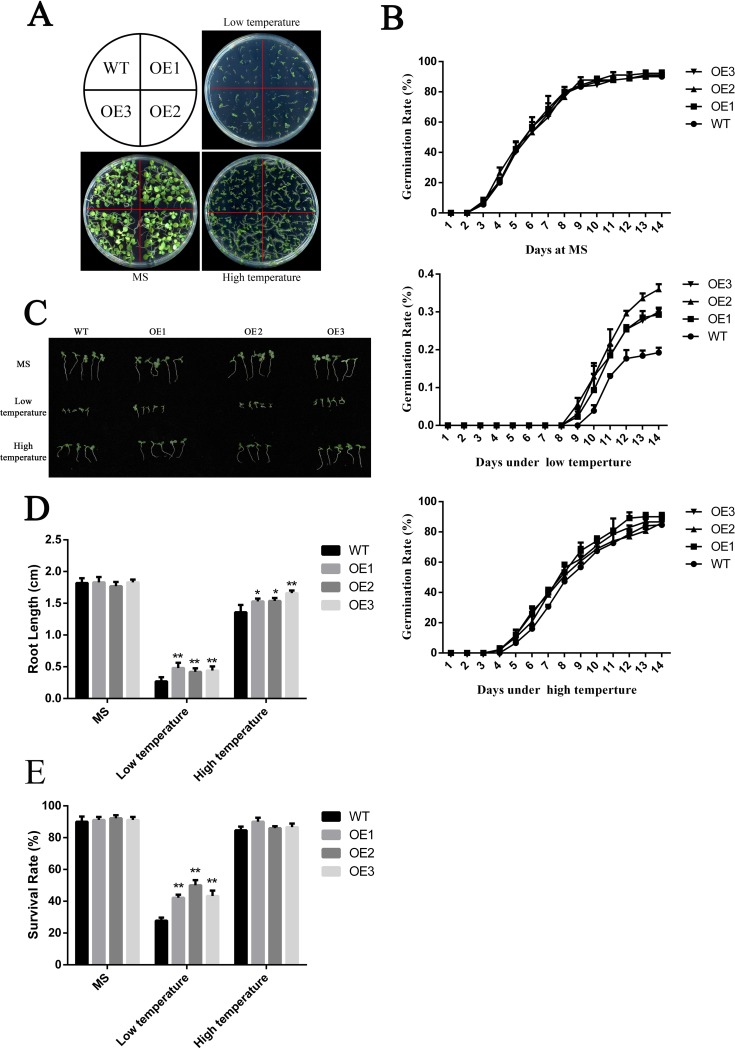
Cold and heat tolerance of wild-type versus *Wrab18*-overexpressing *N*. *benthamiana* plants. **(A)** Phenotypic differences between wild-type and OE lines on MS medium at 18°C and 40°C. **(B)** Germination rate of wild-type and OE lines on MS medium under normal conditions, 18°C, or 40°C. **(C,D)** Root length and **(E)** survival rate of wild-type and OE lines on MS medium under normal conditions, 18°C, or 40°C. Statistically significant differences were analyzed by one-way ANOVA (*p<0.05 or **p<0.01).

To explore the effects of overexpressed WRAB18 in tobacco, we evaluated the developmental conditions and germination of these three transgenic lines. The WT and transgenic tobacco seeds were planted in different area of MS solid medium, each medium contained 0, 100, or 200 mM mannitol respectively and the same concentration gradient of NaCl was setup in other MS solid mediums. There was no visible difference between WT seedlings and transgenic lines under no treatment conditions and only a slight difference under 100 mM NaCl; however, there were more differences under 200 mM mannitol and NaCl treatment. In 200 mM NaCl and mannitol MS medium, the growth of transgenic seedlings was better than that of WT seedlings; this was also observed in plates grown under low temperature (18°C), but the morphology under high temperature (40°C) (Fig 3A) was much better than those under the other treatments (Fig 2B). The germination rate on MS plates were similar, up to 90%, while statistical analysis of the germination rate revealed that ~32% of WT seeds and ~53% of transgenic seeds germinated under 200 mM mannitol after 14 days. Germination rates of OE lines were nearly twice as much as WT plants under 200 mM NaCl treatment (Fig 2C). However, the difference decreased to ~4% at 40°C but reached ~28% at 18°C (Fig 3B).

The transgenic plants also showed stress tolerance in terms of the root length and survival rate. The root growth of the OE transgenic plants was higher than that of the WT, but the phenotype, germination rate, and survival rate were similar. The root length at 40°C was longer than that under other treatments (Figs 2D, 3C and 3D). Meanwhile, except that the survival rate of the transgenic lines was similar to that of the WT at 40°C (Fig 3E), survival rate of transgenic lines was higher than that of the WT under other abiotic stresses (Fig 2E).

### *WRAB18* overexpression enhanced oxidative stress tolerance in plants

**Fig 4:**
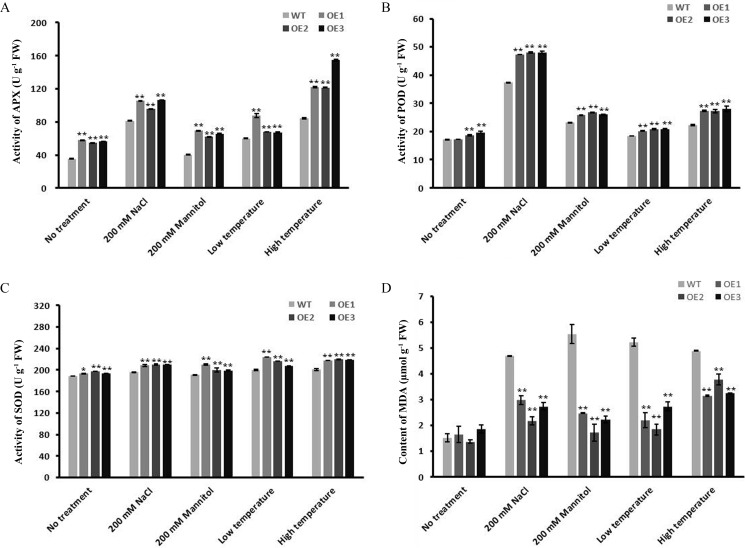
Enzyme activity of antioxidant indexes and MDA content in WT and OE plants under stress. Activity of APX (A), POD (B) and SOD (C), and the MDA content (D) in WT and OE lanes under osmotic stress, high salinity, low and high temperature treatment. Experiments were repeated for 3 times with 10 repetitions for each group. Statistical analysis was performed with one-way ANOVA (*P<0.05, **P<0.001).

When plants suffer from stress, they often show an accelerated ageing process. One obvious observation is a decrease in the activities of enzymes involved in the defense system, such as SOD, APX, and POD [35, 42]. To know the differences in enzymatic activities between WRAB18 overexpression plants and WT lines, the enzymatic activities of APX, POD and SOD, and the content of MDA were detected under both normal and stress conditions (Fig 4). According to the datasets in Fig 4, a little significant differences were observed between WT and transgenic lines before treatments, while all enzymatic activities increased significantly after being treated. However, the changes were quite diverse. The transgenic lines showed a significant increase in APX activities under stress conditions. The activities of POD and SOD in the transgenic tobacco lines were obviously higher than those in the WT lines. MDA content in cell is an important parameter for evaluating the oxidation level of liposomes and the degree of membrane damage [43]. While the MDA content in the WT plants were much higher compared to the transgenic plants after stress treatments. Overall, these data showed that *WRAB18* overexpression resulted in increased antioxidant activity and stability under stress.

### Tissue-specific transcript accumulation and plastid localization of WRAB18

**Fig 5:**
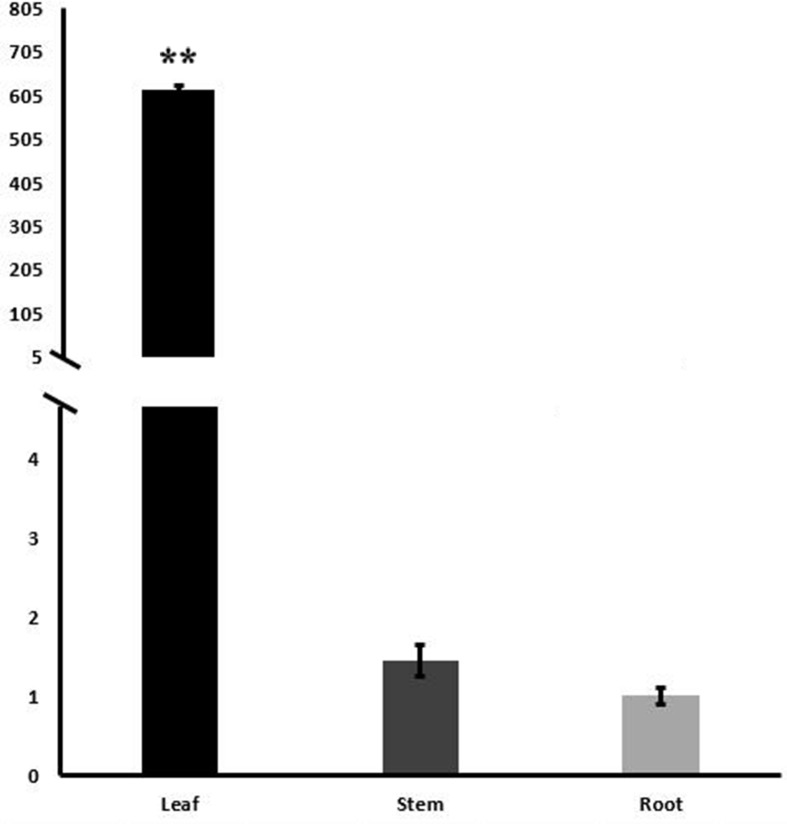
Tissue-specific transcript profiling of *WRAB18* in root, stem and leave. The root, stem and leave samples were collected from two-leaf-period wheat seedlings and qRT-PCR was performed. Data plotted are the mean values ± SD from three independent experiments (n = 3; biological replicates). Statistically significant differences were analyzed using the Student’s t-test (*p<0.05 or **p<0.01).

Real-time quantitative PCR was conducted to investigate the tissue-specific expression patterns of *WRAB18* in two-leaf-period wheat seedlings. The relative expression of *WRAB18* was found in roots, stems and leaves, but strongly expressed in the leaves, the expression level in leaves are almost 4 hundred times and 6 hundred times greater than that in stems and roots, respectively (Fig 5).

**Fig 6:**
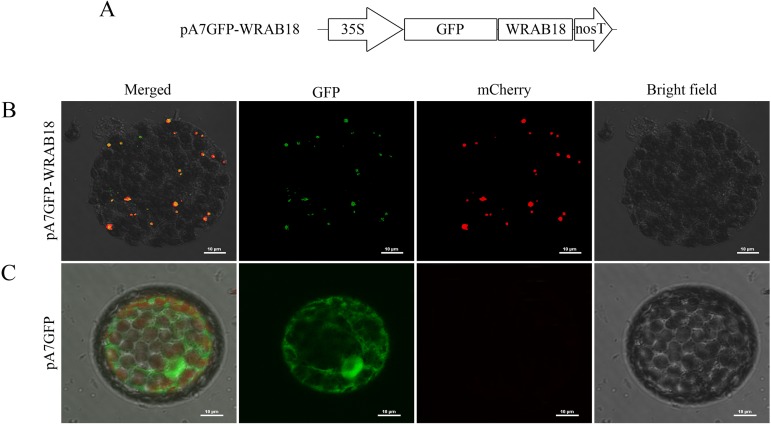
Subcellular localization of WRAB18 in protoplasts of *Nicotiana benthamiana* leaves. **(A)** Schematic representation of recombinant vector pA7GFP::WRAB18. **(B)** Plastid localization of GFP::WRAB18 in protoplasts of *N*. *benthamiana* leaves. Plastid marker protein fluorescence was dispersed in the mCherry channel. GFP::WRAB18 fusion protein fluorescence was merged with the fluorescence emitted by the plastid marker protein. **(C)** Non-specific localization of GFP protein in protoplasts of *N*. *benthamiana* leaves. Images were taken from the eGFP, mCherry channel bright-field, and merged images channels. The bar indicates 10 μm.

GFP, as a reporter molecule, is commonly used for protein expression analyses and as a protein and cell fluorescence tracker to explore protein interactions and conformational changes. Here, a C-terminal GFP recombinant vector was generated to examine subcellular localization of the WRAB18 protein *in vivo* (Fig 6A). The GFP::WRAB18 fusion protein was injected into *N*. *benthamiana* leaves and transiently expressed. The fluorescence signals under the 488 nm GFP channel and mCherry channel (pt-rk CD3-999) were observed in protoplast cells under a confocal microscope and the localization of WRAB18 was found in the plastids of protoplast cells, as shown in Fig 6B.

### WRAB18 protected LDH enzyme activity under stress

Many LEA proteins prevent protein aggregation [44, 45]. LDH is a key enzyme of the glycolytic pathway and can lead to mutual conversion between lactic acid and pyruvate. LDH is stable under normal condition, but under stress, such as freezing and high temperatures, drought, and salinity, the activity quickly decreases. LDH is considered as a model enzyme to detect the protective functions of proteins based on enzyme activity [46].

**Fig 7:**
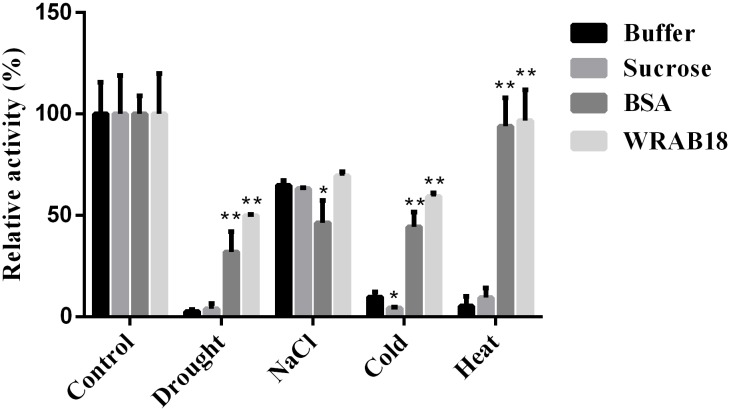
Protective function of WRAB18 on LDH activity under severe conditions. LDH solution was cultured with BSA, sucrose solution, and PBS buffer, and purified WRAB18 was dehydrated to 70%, rehydrated to the original volume, supplemented with 200 mM NaCl for 2 h, placed on ice for 12 h, and left at 45°**C** for 30 min. Reactions were repeated three times. Statistically significant differences were analyzed using Student’s t-test (*p<0.05 or **p<0.01).

In this experiment, BSA and sucrose solution were used as positive and negative controls, respectively, to assess the protective effects of WRAB18 on LDH. The activity of LDH was defined as 100% in the absence of stress. After treatment under various stress conditions for different times, noticeable differences were observed among the four types of stress. WRAB18 showed greater protection of LDH activity during drought, cold, and heat stress. More than 80% of LDH activity was preserved and was slightly higher when that was conferred by BSA under heat, while the sucrose solution and buffer did not preserve LDH activity. In addition, sucrose is worse than buffer in the cold experiment. Interestingly, buffer and sucrose exhibited greater protection than BSA did, but protection was still weaker than that conferred by WRAB18 under high salinity treatment (Fig 7). Overall, our data indicated that WRAB18 possesses a protective effect towards LDH *in vitro* against adverse stresses including drought, high salinity, and low and high temperatures.

## Discussion

As typical stress-response proteins, LEA proteins also always play roles in plants under various stress conditions [1, 47]. Previous study showed that WRAB18, as a member of the LEA protein family, functions as a downstream response factor involving the ABA-dependent signaling pathway [32]. However, none of the others stress-response abilities were elucidated, while proteins in group 3 LEA family are widely considered to be multifunctional. In this report, we performed *in vivo* and *in vitro* functional analysis to explore the protective effects of WRAB18 in different species cells under four kinds of stress (osmotic stress, high salinity, low and high temperature). Our results suggested that WRAB18 enhances the growth of *E*. *coli*, confers abiotic stress tolerance to transgenic tobacco, is localized in the plastids of plant cells, and protects the enzymatic activity of LDH under severe conditions.

Heterologous expression has been frequently-used to confirm a protein’s function *in vivo*. Here, both *E*.*coli* protein express system and transgenic approach were utilized to detect the functions of WRAB18 in prokaryotic and eukaryotic cells under four different abiotic stress treatments. The growth condition of *E*. *coli* harboring the recombinant WRAB18 protein exhibited greater tolerance to stress than the control did, which contained only the empty plasmid vector. Previous reports have declared that overexpression of LEA proteins from different species in *Arabidopsis*, *N*. *benthamiana*, rice and other plants reveal greater growth during stress treatments [48], which was congruence with our results. The growth and morphology of *WRAB18*-transgenic plants were much better than those of the WT lines under abiotic stress (osmotic stress, high salinity, low and high temperature), based on the data of phenotypic observations, the germination rate, survival rate, and root length. Environmental stress always leads to the excessive accumulation of reactive oxygen species (ROS) in plant cells, which directly results in the nucleic acid mutation and fracture, the change of protein conformation and cell membrane damage [49]. The existence of antioxidants is essential for cells to defense these damages. The activities of stress-related antioxidant enzymes (APX, POD and SOD) in the *WRAB18*-transgenic tobacco lines were higher during stresses. Damage caused by ROS leads to the oxidation of liposomes results in the increase of MDA levels [50]. In our datasets, *WRAB18*-transgenic plants revealed a lower level of MDA compared with WT lines during stress conditions. All these data demonstrated that the enhanced tolerance of the *WRAB18*-transgenic lines under stresses could be attributed to the overexpression of WRAB18 protein.

Stress also lead to metabolic disorders in cells. Many signaling pathways are activated, and stress tolerance is important for maintaining the balance among cell components to minimize potential damage. Proteins with highly hydrophilic amino acids are reported to protect enzyme activities under stress [46, 51]. Group 3 LEA proteins are not classical chaperones but are thought to function as molecular chaperones that stabilize functional proteins under stress [52]. Based on the assessment criteria for protective function, LDH activity was measured after being incubated with WRAB18 protein or other control buffer under stresses (osmotic stress, high salinity, low and high temperature). Consistent with the previously reported results, WRAB18 did show the protective effect on LDH enzyme activity. Researches by Battaglia et al. (2008) suggests that LEA proteins can provide an environment with relative sufficient moisture for the target enzymes to maintain their completed structure and avoid the passivation and inactivation under stresses [14]. The higher hydrophilic α-helix and greater percentage of hydrophilic amino acids in WRAB18 sequence was a solid structure foundation for this protective function. However, the protective effect of WRAB18 on LDH under high salinity treatment was not so much remarkable than the other reactions. That was because LDH is more active in the presence of salt, that is to say LDH has certain salinity resistance to some extent [53]. So that the activity of LDH under NaCl treatment was higher than that during drought stress, and the buffer condition seemed not vital, WRAB18 did not show greater advantages than the other solution.

LEA proteins exhibit diverse subcellular localization patterns, which are related to their multiple functions [54, 55]. Many group 3 LEA proteins have been found distributed within cytoplasm in earlier studies [48]. However, based on further studies, more different subcellular localizations of group 3 LEA proteins were observed. PsLEAm from pea showed localization in mitochondria [56], while LEA3-L2 and WCS19 from wheat were localized in the chloroplast [57]. WAP27A and WAP27B are abundantly accumulated in endoplasmic reticulum of cortical parenchyma cells of the mulberry tree (*Morus bombycis*) [58]. To investigate the functions of WRAB18 intracellularly, subcellular localization experiments were designed to investigate the localization of the protein *in vivo*. The WRAB18::GFP fusion protein was transferred into tobacco leaves together with a plastid protein marker. The merged fluorescent signals showed that WRAB18 localizes in plastids, which exist in young cells and play a role in organization of epidermal cells, as well as in manufacturing and storage of starch, protein, and fat [59]. This observation increases our understanding of the role of WRAB18.

Carbohydrate metabolism in cells is crucial in both prokaryotes and eukaryotes. Carbohydrates are not only the primary components of cell structures but also provide energetic materials for organisms and play important roles in regulating the cell activities [60–62]. The highly hydrophilic group 3 LEA proteins form a randomly coiled structure in solution; however, the simulation experiments *in vitro* showed that LEA proteins form a tight hydrogen bonding network in dehydrated cells together with sugars to confer long-term stability to the sugar [63]. In other words, sugar can affect the structure of group 3 LEA proteins. Structures of group 3 LEA proteins can change from the disordered state into a structure containing α-helixes under the interference of sugar during stress conditions. This structure contributes to group 3 LEA proteins and plays a role in protecting other molecules from stress [14]. Like other group 3 LEA proteins, WRAB18, localizes in plastids, can also form hydrophilic α-helixes to protect the functional proteins within the mediation of carbohydrate in plastids. The protective function was performed just like what it did to LDH.

In conclusion, the tolerance assays of *E*. *coli* and transgenic tobacco confirmed that the WRAB18 protein responds not only to ABA, but obviously to three types of stress (osmotic stress, high salinity, and low temperature), and indistinctively to high temperature. Considering its plastid localization and protective effect on enzyme activities, we conclude that WRAB18 responds to different stresses in both prokaryotic and eukaryotic species and acts like a “molecular shield” to protect the activities of enzymes under stress conditions. However, the complex regulatory mechanisms involving WRAB18 are still unclear, more details are required to be deciphered.

## Supporting information

S1 Fig Sequence analysis and three-dimensional structure prediction of WRAB18. **(A)** Multiple amino acid sequence alignment of TaWRAB18 (BAC80266.1) with TaWRAB17 (BAF79926.1), TaWRAB19 (AAF68627.1), TaLEA3 (AAN74639.1) and AcLEA3 (ADC55280.1). The conserved sequences are indicated in the boxes. Identical amino acids are shaded in red. The predicted structural domains of alpha-helicies are showed with helical line **(B)** The three-dimensional structure prediction of WRAB18.
(TIF)

S2 Fig SDS-PAGE of overexpressed WRAB18 in BL21 and purified WRAB18. **(A)** SDS-PAGE stained by Coomassie Brilliant Blue show the expression of WRAB18; the target band represents the protein fused to a 6×His tag migrated at approximately 21 kDa. Band 1 represents the overexpressing WRAB18 induced by IPTG. *E*. *coli* harboring only the empty pET28a vector is served as the control, showed in band 2. Band 3 indicates the purified WRAB18. The band M represents the protein marker. **(B)** The immunodetection result of the expression and purified WRAB18 using anti-His-tag rabbit polyclonal antibody. Lanes 4, 5, 6 represent the immunodetective signal band of empty pET28a control, the overexpressing WRAB18 and the purified WRAB18, respectively.
(TIF)

S1 Table Primers used in this study. (DOCX)
