# Supplementary figures and images for: Triticum aestivum WRAB18 functions in plastids and confers abiotic stress tolerance when overexpressed in Escherichia coli and Nicotiania benthamiana
Source: PLoS One. 2017 Feb 16;12(2):e0171340. doi: 10.1371/journal.pone.0171340 (PMC5313140; doi:10.1371/journal.pone.0171340)

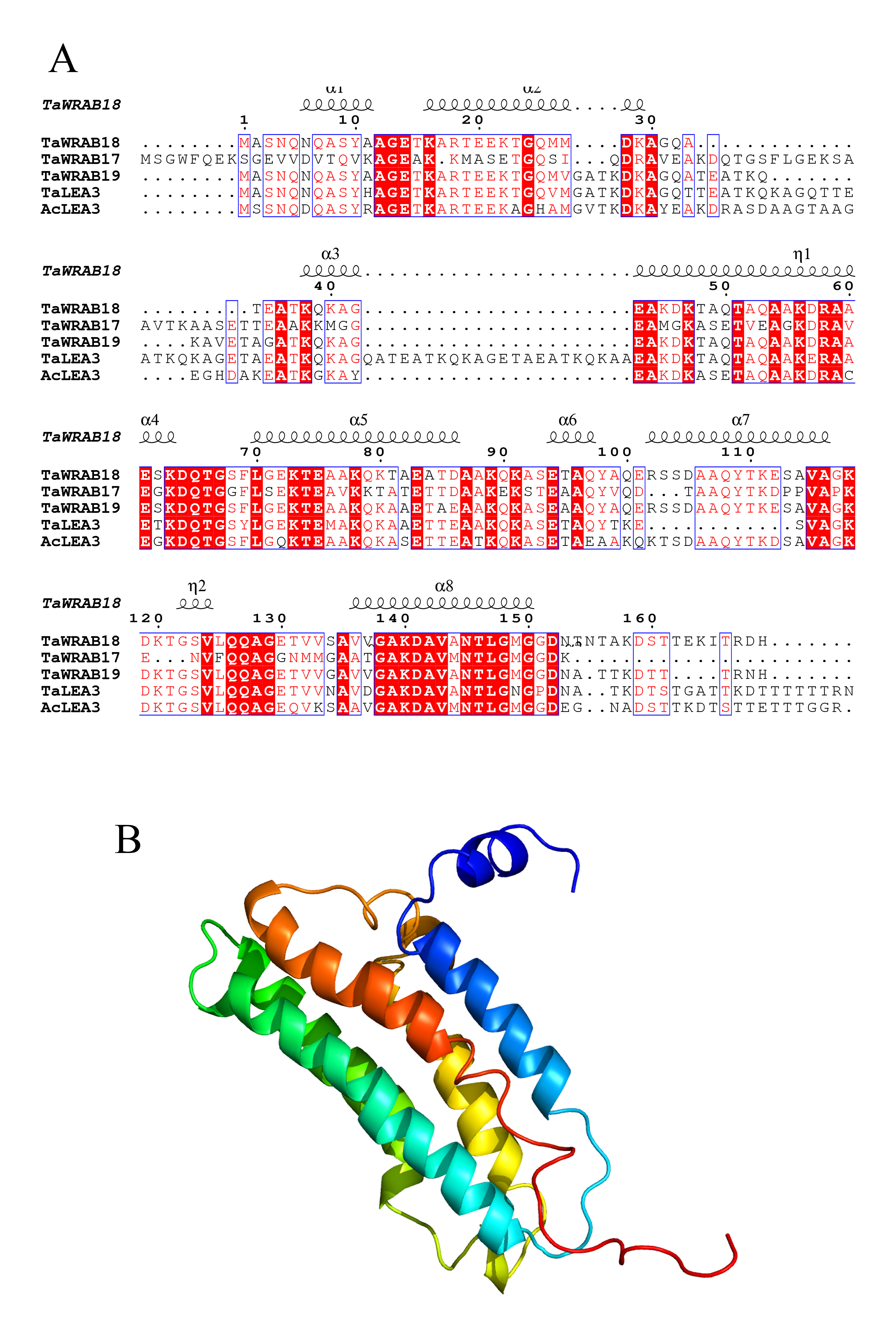

Supplement: S1 Fig — (A) Multiple amino acid sequence alignment of TaWRAB18 (BAC80266.1) with TaWRAB17 (BAF79926.1), TaWRAB19 (AAF68627.1), TaLEA3 (AAN74639.1) and AcLEA3 (ADC55280.1). The conserved sequences are indicated in the boxes. Identical amino acids are shaded in red. The predicted structural domains of alpha-helicies are showed with helical line (B) The three-dimensional structure prediction of WRAB18. (TIF) [file pone.0171340.s001.tif]

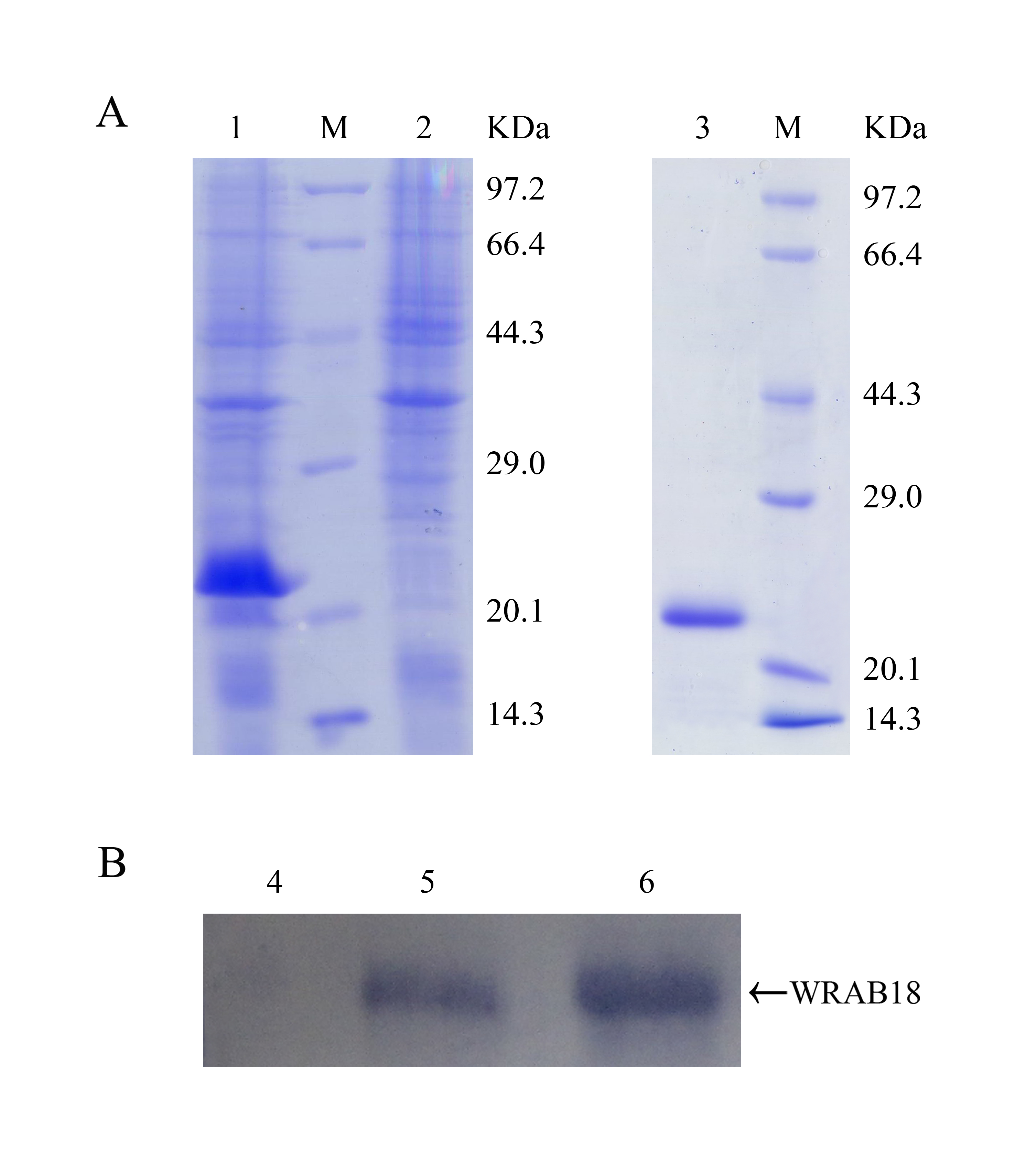

Supplement: S2 Fig — (A) SDS-PAGE stained by Coomassie Brilliant Blue show the expression of WRAB18; the target band represents the protein fused to a 6×His tag migrated at approximately 21 kDa. Band 1 represents the overexpressing WRAB18 induced by IPTG. E. coli harboring only the empty pET28a vector is served as the control, showed in band 2. Band 3 indicates the purified WRAB18. The band M represents the protein marker. (B) The immunodetection result of the expression and purified WRAB18 using anti-His-tag rabbit polyclonal antibody. Lanes 4, 5, 6 represent the immunodetective signal band of empty pET28a control, the overexpressing WRAB18 and the purified WRAB18, respectively. (TIF) [file pone.0171340.s002.tif]
