# Supplementary material for: Triticum aestivum WRAB18 functions in plastids and confers abiotic stress tolerance when overexpressed in Escherichia coli and Nicotiania benthamiana
Source: PLoS One. 2017 Feb 16;12(2):e0171340. doi: 10.1371/journal.pone.0171340 (PMC5313140; doi:10.1371/journal.pone.0171340)
Supplement: S1 Table — (DOCX) [file pone.0171340.s003.docx]

**S1 Table. Primers used in this study.**

| ***Primer name*** | ***primer sequence(5'→3')*** |
| --- | --- |
| *WRAB18* F | ATGGCCTCCAACCAGAACCAGGCGAG |
| *WRAB18* R | CTAGTGATCCCTAGTGATCTTCTCGG |
| Expression Primer F | GCGAATTCATGGCCTCCAACCAGAACC |
| Expression Primer R | CCCAAGCTTCTAGTGATCCCTAGTGATCT |
| Transgenic Primer F | GTACTCTAGAATGGCCTCCAACCAGAACC |
| Transgenic Primer R  Real-Time PCR Primer F  Real-Time PCR Primer R | CGGAGCTCCTAGTGATCCCTAGTGATCT  GGACAAGGCGGGGCAGG  CCGCATCGGTTGCCTCG |
| Sublocalization Primer F | CTAGTCTAGAATGGCCTCCAACCAGAACC |
| Sublocalization Primer R | TCCCCCGGGCTAGTGATCCCTAGTGATCT |
| *β-actin* F | TGGACTCTGGTGATGGTGTC |
| *β-actin* R | CCTCCAATCCAAACACTGTA |

Restriction sites are underlined.
